# Supplementary material for: Pyruvate Dehydrogenase Kinase 1 inhibition mediated oxidative phosphorylation enhancement in cartilage promotes osteoarthritis progression
Source: BMC Musculoskelet Disord. 2023 Jul 20;24:597. doi: 10.1186/s12891-023-06585-6 (PMC10357736; doi:10.1186/s12891-023-06585-6)
Supplement: Supplementary file 1 — Additional file 1. [file 12891_2023_6585_MOESM1_ESM.zip › KEGG.Gsea.1653622432464/KEGG_TOLL_LIKE_RECEPTOR_SIGNALING_PATHWAY.html]

Details for gene set KEGG\_TOLL\_LIKE\_RECEPTOR\_SIGNALING\_PATHWAY[GSEA]

|  || Dataset | 55457\_collapsed\_to\_symbols.cls.cls #OA\_versus\_normal.cls.cls #OA\_versus\_normal\_repos |
| Phenotype | cls.cls#OA\_versus\_normal\_repos |
| Upregulated in class | normal |
| GeneSet | KEGG\_TOLL\_LIKE\_RECEPTOR\_SIGNALING\_PATHWAY |
| Enrichment Score (ES) | -0.2717023 |
| Normalized Enrichment Score (NES) | -1.1733133 |
| Nominal p-value | 0.18367347 |
| FDR q-value | 0.41389096 |
| FWER p-Value | 1.0 |
Table: GSEA Results Summary

  

Fig 1: Enrichment plot: KEGG\_TOLL\_LIKE\_RECEPTOR\_SIGNALING\_PATHWAY      
 Profile of the Running ES Score & Positions of GeneSet Members on the Rank Ordered List

  

| SYMBOL | TITLE | RANK IN GENE LIST | RANK METRIC SCORE | RUNNING ES | CORE ENRICHMENT || 1 | TLR7 | toll like receptor 7 [Source:HGNC Symbol;Acc:HGNC:15631] | 61 | 0.971 | 0.0270 | No |
| 2 | TLR3 | toll like receptor 3 [Source:HGNC Symbol;Acc:HGNC:11849] | 175 | 0.837 | 0.0456 | No |
| 3 | IFNAR2 | interferon alpha and beta receptor subunit 2 [Source:HGNC Symbol;Acc:HGNC:5433] | 616 | 0.618 | 0.0315 | No |
| 4 | CD86 | CD86 molecule [Source:HGNC Symbol;Acc:HGNC:1705] | 689 | 0.596 | 0.0454 | No |
| 5 | MAPK9 | mitogen-activated protein kinase 9 [Source:HGNC Symbol;Acc:HGNC:6886] | 721 | 0.588 | 0.0622 | No |
| 6 | MAPK3 | mitogen-activated protein kinase 3 [Source:HGNC Symbol;Acc:HGNC:6877] | 757 | 0.579 | 0.0785 | No |
| 7 | TAB2 | TGF-beta activated kinase 1 (MAP3K7) binding protein 2 [Source:HGNC Symbol;Acc:HGNC:17075] | 877 | 0.549 | 0.0871 | No |
| 8 | FADD | Fas associated via death domain [Source:HGNC Symbol;Acc:HGNC:3573] | 924 | 0.534 | 0.1011 | No |
| 9 | AKT1 | AKT serine/threonine kinase 1 [Source:HGNC Symbol;Acc:HGNC:391] | 1192 | 0.478 | 0.0959 | No |
| 10 | IFNA8 | interferon alpha 8 [Source:HGNC Symbol;Acc:HGNC:5429] | 1206 | 0.475 | 0.1104 | No |
| 11 | MAP3K7 | mitogen-activated protein kinase kinase kinase 7 [Source:HGNC Symbol;Acc:HGNC:6859] | 1701 | 0.399 | 0.0849 | No |
| 12 | CXCL10 | C-X-C motif chemokine ligand 10 [Source:HGNC Symbol;Acc:HGNC:10637] | 1709 | 0.396 | 0.0973 | No |
| 13 | CD14 | CD14 molecule [Source:HGNC Symbol;Acc:HGNC:1628] | 1928 | 0.369 | 0.0924 | No |
| 14 | TNF | tumor necrosis factor [Source:HGNC Symbol;Acc:HGNC:11892] | 2098 | 0.350 | 0.0906 | No |
| 15 | PIK3CB | "phosphatidylinositol-4,5-bisphosphate 3-kinase catalytic subunit beta [Source:HGNC Symbol;Acc:HGNC:8976]" | 2186 | 0.340 | 0.0950 | No |
| 16 | MAP2K4 | mitogen-activated protein kinase kinase 4 [Source:HGNC Symbol;Acc:HGNC:6844] | 2214 | 0.337 | 0.1039 | No |
| 17 | MAPK14 | mitogen-activated protein kinase 14 [Source:HGNC Symbol;Acc:HGNC:6876] | 2352 | 0.320 | 0.1037 | No |
| 18 | MAPK13 | mitogen-activated protein kinase 13 [Source:HGNC Symbol;Acc:HGNC:6875] | 2544 | 0.298 | 0.0985 | No |
| 19 | TLR1 | toll like receptor 1 [Source:HGNC Symbol;Acc:HGNC:11847] | 3049 | 0.245 | 0.0672 | No |
| 20 | TLR5 | toll like receptor 5 [Source:HGNC Symbol;Acc:HGNC:11851] | 3261 | 0.225 | 0.0581 | No |
| 21 | CXCL11 | C-X-C motif chemokine ligand 11 [Source:HGNC Symbol;Acc:HGNC:10638] | 3575 | 0.196 | 0.0401 | No |
| 22 | TLR8 | toll like receptor 8 [Source:HGNC Symbol;Acc:HGNC:15632] | 3579 | 0.196 | 0.0462 | No |
| 23 | IFNAR1 | interferon alpha and beta receptor subunit 1 [Source:HGNC Symbol;Acc:HGNC:5432] | 3587 | 0.195 | 0.0521 | No |
| 24 | LY96 | lymphocyte antigen 96 [Source:HGNC Symbol;Acc:HGNC:17156] | 3630 | 0.191 | 0.0550 | No |
| 25 | RELA | "RELA proto-oncogene, NF-kB subunit [Source:HGNC Symbol;Acc:HGNC:9955]" | 3942 | 0.161 | 0.0360 | No |
| 26 | SPP1 | secreted phosphoprotein 1 [Source:HGNC Symbol;Acc:HGNC:11255] | 4205 | 0.137 | 0.0201 | No |
| 27 | IFNA4 | interferon alpha 4 [Source:HGNC Symbol;Acc:HGNC:5425] | 4282 | 0.129 | 0.0184 | No |
| 28 | IRF7 | interferon regulatory factor 7 [Source:HGNC Symbol;Acc:HGNC:6122] | 4411 | 0.120 | 0.0123 | No |
| 29 | STAT1 | signal transducer and activator of transcription 1 [Source:HGNC Symbol;Acc:HGNC:11362] | 4495 | 0.113 | 0.0095 | No |
| 30 | NFKB1 | nuclear factor kappa B subunit 1 [Source:HGNC Symbol;Acc:HGNC:7794] | 4626 | 0.102 | 0.0027 | No |
| 31 | CTSK | cathepsin K [Source:HGNC Symbol;Acc:HGNC:2536] | 4869 | 0.082 | -0.0135 | No |
| 32 | TAB1 | TGF-beta activated kinase 1 (MAP3K7) binding protein 1 [Source:HGNC Symbol;Acc:HGNC:18157] | 5684 | 0.017 | -0.0765 | No |
| 33 | TRAF6 | TNF receptor associated factor 6 [Source:HGNC Symbol;Acc:HGNC:12036] | 5761 | 0.011 | -0.0821 | No |
| 34 | IFNA2 | interferon alpha 2 [Source:HGNC Symbol;Acc:HGNC:5423] | 5869 | 0.004 | -0.0903 | No |
| 35 | IKBKB | inhibitor of nuclear factor kappa B kinase subunit beta [Source:HGNC Symbol;Acc:HGNC:5960] | 5914 | 0.001 | -0.0937 | No |
| 36 | IL12B | interleukin 12B [Source:HGNC Symbol;Acc:HGNC:5970] | 5991 | -0.004 | -0.0995 | No |
| 37 | TRAF3 | TNF receptor associated factor 3 [Source:HGNC Symbol;Acc:HGNC:12033] | 6108 | -0.013 | -0.1081 | No |
| 38 | IFNA21 | interferon alpha 21 [Source:HGNC Symbol;Acc:HGNC:5424] | 6127 | -0.015 | -0.1091 | No |
| 39 | TOLLIP | toll interacting protein [Source:HGNC Symbol;Acc:HGNC:16476] | 6438 | -0.038 | -0.1320 | No |
| 40 | CCL5 | C-C motif chemokine ligand 5 [Source:HGNC Symbol;Acc:HGNC:10632] | 6584 | -0.048 | -0.1418 | No |
| 41 | MYD88 | MYD88 innate immune signal transduction adaptor [Source:HGNC Symbol;Acc:HGNC:7562] | 6638 | -0.052 | -0.1442 | No |
| 42 | IFNA10 | interferon alpha 10 [Source:HGNC Symbol;Acc:HGNC:5418] | 6682 | -0.054 | -0.1458 | No |
| 43 | IFNA5 | interferon alpha 5 [Source:HGNC Symbol;Acc:HGNC:5426] | 6755 | -0.059 | -0.1494 | No |
| 44 | CD40 | CD40 molecule [Source:HGNC Symbol;Acc:HGNC:11919] | 6825 | -0.065 | -0.1527 | No |
| 45 | MAPK1 | mitogen-activated protein kinase 1 [Source:HGNC Symbol;Acc:HGNC:6871] | 6853 | -0.068 | -0.1526 | No |
| 46 | CD80 | CD80 molecule [Source:HGNC Symbol;Acc:HGNC:1700] | 6918 | -0.074 | -0.1552 | No |
| 47 | IL12A | interleukin 12A [Source:HGNC Symbol;Acc:HGNC:5969] | 6930 | -0.074 | -0.1536 | No |
| 48 | CXCL9 | C-X-C motif chemokine ligand 9 [Source:HGNC Symbol;Acc:HGNC:7098] | 7024 | -0.082 | -0.1582 | No |
| 49 | RAC1 | Rac family small GTPase 1 [Source:HGNC Symbol;Acc:HGNC:9801] | 7192 | -0.096 | -0.1681 | No |
| 50 | MAP2K6 | mitogen-activated protein kinase kinase 6 [Source:HGNC Symbol;Acc:HGNC:6846] | 7246 | -0.099 | -0.1690 | No |
| 51 | IFNA6 | interferon alpha 6 [Source:HGNC Symbol;Acc:HGNC:5427] | 7272 | -0.101 | -0.1677 | No |
| 52 | PIK3CD | "phosphatidylinositol-4,5-bisphosphate 3-kinase catalytic subunit delta [Source:HGNC Symbol;Acc:HGNC:8977]" | 7405 | -0.111 | -0.1743 | No |
| 53 | TLR4 | toll like receptor 4 [Source:HGNC Symbol;Acc:HGNC:11850] | 7703 | -0.134 | -0.1931 | No |
| 54 | PIK3CA | "phosphatidylinositol-4,5-bisphosphate 3-kinase catalytic subunit alpha [Source:HGNC Symbol;Acc:HGNC:8975]" | 7733 | -0.136 | -0.1909 | No |
| 55 | PIK3CG | "phosphatidylinositol-4,5-bisphosphate 3-kinase catalytic subunit gamma [Source:HGNC Symbol;Acc:HGNC:8978]" | 7953 | -0.152 | -0.2031 | No |
| 56 | TLR2 | toll like receptor 2 [Source:HGNC Symbol;Acc:HGNC:11848] | 7991 | -0.154 | -0.2009 | No |
| 57 | AKT3 | AKT serine/threonine kinase 3 [Source:HGNC Symbol;Acc:HGNC:393] | 8008 | -0.156 | -0.1971 | No |
| 58 | MAPK12 | mitogen-activated protein kinase 12 [Source:HGNC Symbol;Acc:HGNC:6874] | 8161 | -0.168 | -0.2034 | No |
| 59 | IFNA7 | interferon alpha 7 [Source:HGNC Symbol;Acc:HGNC:5428] | 8533 | -0.196 | -0.2260 | No |
| 60 | IFNA1 | interferon alpha 1 [Source:HGNC Symbol;Acc:HGNC:5417] | 8640 | -0.204 | -0.2276 | No |
| 61 | PIK3R3 | phosphoinositide-3-kinase regulatory subunit 3 [Source:HGNC Symbol;Acc:HGNC:8981] | 8801 | -0.217 | -0.2330 | No |
| 62 | PIK3R5 | phosphoinositide-3-kinase regulatory subunit 5 [Source:HGNC Symbol;Acc:HGNC:30035] | 8804 | -0.217 | -0.2260 | No |
| 63 | MAPK8 | mitogen-activated protein kinase 8 [Source:HGNC Symbol;Acc:HGNC:6881] | 8956 | -0.228 | -0.2304 | No |
| 64 | IRAK1 | interleukin 1 receptor associated kinase 1 [Source:HGNC Symbol;Acc:HGNC:6112] | 9377 | -0.262 | -0.2546 | No |
| 65 | TICAM1 | toll like receptor adaptor molecule 1 [Source:HGNC Symbol;Acc:HGNC:18348] | 9569 | -0.280 | -0.2603 | Yes |
| 66 | IKBKE | inhibitor of nuclear factor kappa B kinase subunit epsilon [Source:HGNC Symbol;Acc:HGNC:14552] | 9646 | -0.287 | -0.2569 | Yes |
| 67 | IRF5 | interferon regulatory factor 5 [Source:HGNC Symbol;Acc:HGNC:6120] | 9688 | -0.290 | -0.2506 | Yes |
| 68 | MAPK10 | mitogen-activated protein kinase 10 [Source:HGNC Symbol;Acc:HGNC:6872] | 9699 | -0.291 | -0.2418 | Yes |
| 69 | LBP | lipopolysaccharide binding protein [Source:HGNC Symbol;Acc:HGNC:6517] | 9769 | -0.298 | -0.2374 | Yes |
| 70 | MAP2K2 | mitogen-activated protein kinase kinase 2 [Source:HGNC Symbol;Acc:HGNC:6842] | 9932 | -0.315 | -0.2398 | Yes |
| 71 | MAP2K1 | mitogen-activated protein kinase kinase 1 [Source:HGNC Symbol;Acc:HGNC:6840] | 10197 | -0.340 | -0.2493 | Yes |
| 72 | CASP8 | caspase 8 [Source:HGNC Symbol;Acc:HGNC:1509] | 10442 | -0.366 | -0.2563 | Yes |
| 73 | FOS | "Fos proto-oncogene, AP-1 transcription factor subunit [Source:HGNC Symbol;Acc:HGNC:3796]" | 10640 | -0.387 | -0.2590 | Yes |
| 74 | IFNB1 | interferon beta 1 [Source:HGNC Symbol;Acc:HGNC:5434] | 10801 | -0.406 | -0.2582 | Yes |
| 75 | IRAK4 | interleukin 1 receptor associated kinase 4 [Source:HGNC Symbol;Acc:HGNC:17967] | 10874 | -0.413 | -0.2503 | Yes |
| 76 | IRF3 | interferon regulatory factor 3 [Source:HGNC Symbol;Acc:HGNC:6118] | 10879 | -0.414 | -0.2371 | Yes |
| 77 | TLR6 | toll like receptor 6 [Source:HGNC Symbol;Acc:HGNC:16711] | 10941 | -0.423 | -0.2280 | Yes |
| 78 | PIK3R2 | phosphoinositide-3-kinase regulatory subunit 2 [Source:HGNC Symbol;Acc:HGNC:8980] | 10956 | -0.425 | -0.2152 | Yes |
| 79 | IKBKG | inhibitor of nuclear factor kappa B kinase regulatory subunit gamma [Source:HGNC Symbol;Acc:HGNC:5961] | 10986 | -0.428 | -0.2034 | Yes |
| 80 | PIK3R1 | phosphoinositide-3-kinase regulatory subunit 1 [Source:HGNC Symbol;Acc:HGNC:8979] | 11099 | -0.443 | -0.1976 | Yes |
| 81 | CCL4 | C-C motif chemokine ligand 4 [Source:HGNC Symbol;Acc:HGNC:10630] | 11211 | -0.458 | -0.1913 | Yes |
| 82 | TBK1 | TANK binding kinase 1 [Source:HGNC Symbol;Acc:HGNC:11584] | 11326 | -0.474 | -0.1847 | Yes |
| 83 | CCL3 | C-C motif chemokine ligand 3 [Source:HGNC Symbol;Acc:HGNC:10627] | 11465 | -0.497 | -0.1792 | Yes |
| 84 | MAPK11 | mitogen-activated protein kinase 11 [Source:HGNC Symbol;Acc:HGNC:6873] | 11501 | -0.501 | -0.1655 | Yes |
| 85 | CHUK | component of inhibitor of nuclear factor kappa B kinase complex [Source:HGNC Symbol;Acc:HGNC:1974] | 11624 | -0.521 | -0.1579 | Yes |
| 86 | RIPK1 | receptor interacting serine/threonine kinase 1 [Source:HGNC Symbol;Acc:HGNC:10019] | 11953 | -0.584 | -0.1644 | Yes |
| 87 | MAP3K8 | mitogen-activated protein kinase kinase kinase 8 [Source:HGNC Symbol;Acc:HGNC:6860] | 11975 | -0.589 | -0.1468 | Yes |
| 88 | MAP2K3 | mitogen-activated protein kinase kinase 3 [Source:HGNC Symbol;Acc:HGNC:6843] | 12004 | -0.593 | -0.1296 | Yes |
| 89 | IL1B | interleukin 1 beta [Source:HGNC Symbol;Acc:HGNC:5992] | 12105 | -0.622 | -0.1170 | Yes |
| 90 | MAP2K7 | mitogen-activated protein kinase kinase 7 [Source:HGNC Symbol;Acc:HGNC:6847] | 12209 | -0.649 | -0.1038 | Yes |
| 91 | CXCL8 | C-X-C motif chemokine ligand 8 [Source:HGNC Symbol;Acc:HGNC:6025] | 12297 | -0.676 | -0.0885 | Yes |
| 92 | IL6 | interleukin 6 [Source:HGNC Symbol;Acc:HGNC:6018] | 12460 | -0.730 | -0.0772 | Yes |
| 93 | JUN | "Jun proto-oncogene, AP-1 transcription factor subunit [Source:HGNC Symbol;Acc:HGNC:6204]" | 12813 | -0.994 | -0.0721 | Yes |
| 94 | NFKBIA | NFKB inhibitor alpha [Source:HGNC Symbol;Acc:HGNC:7797] | 12817 | -1.001 | -0.0396 | Yes |
| 95 | AKT2 | AKT serine/threonine kinase 2 [Source:HGNC Symbol;Acc:HGNC:392] | 12896 | -1.404 | 0.0003 | Yes |
Table: GSEA details [plain text format]

  

Fig 2: KEGG\_TOLL\_LIKE\_RECEPTOR\_SIGNALING\_PATHWAY      
 Blue-Pink O' Gram in the Space of the Analyzed GeneSet

  

Fig 3: KEGG\_TOLL\_LIKE\_RECEPTOR\_SIGNALING\_PATHWAY: Random ES distribution      
 Gene set null distribution of ES for **KEGG\_TOLL\_LIKE\_RECEPTOR\_SIGNALING\_PATHWAY**
